# Multi‐domain intergenerational programming for brain health: protocol for a well‐being focused dementia risk reduction pilot trial

**DOI:** 10.1002/alz70860_100685

**Published:** 2025-12-23

**Authors:** April Mallon, Mei Ling Lim, Shannon Jarrott, Amy Sparks, Katie Harris, Amelia Trippas, Catherine Ho, Christina Coleman, Ebony Lewis, Nicole Ee, Rachelle Scott, Sarah Kedwell, Ruth Peters

**Affiliations:** ^1^ University of New South Wales, Sydney, NSW, Australia; ^2^ The George Institute for Global Health, Sydney, NSW, Australia; ^3^ Ohio State University, Columbus, OH, USA; ^4^ George Institute for Global Health, UNSW, Sydney, NSW, Australia; ^5^ Neuroscience Research Australia, Sydney, NSW, Australia; ^6^ The George Institute for Global Health, University of New South Wales, Imperial College London, Sydney, NSW, Australia

## Abstract

**Background:**

Recent evidence identifies that Positive Mental Health (PMH) factors such as purpose in life, life satisfaction, and emotional well‐being, may protect against cognitive decline and dementia. Interventions aiming to enhance PMH (“Positive Psychological Interventions”) have also been shown to improve engagement in behaviours protective for brain‐health such as good nutrition, smoking cessation, and adherence to hypertension treatment. These positive‐psychological interventions (PPIs) – often involving skills such as gratitude, mindfulness, and cognitive restructuring –may be a valuable addition to multi‐domain interventions for dementia prevention by bolstering effectiveness and promoting sustained change. However, while evidence suggests the benefits of PPIs for risk factor modification, it remains unclear whether this will improve cognitive outcomes. Therefore, GenWell‐BRAVE aims to (1) evaluate the feasibility of prescribing PPI within a multi‐domain intergenerational program and (2) estimate relative program efficacy for improving dementia risk profiles of older adults compared to an intergenerational program without PPI.

**Method:**

GenWell‐BRAVE is a sub‐study of The INTEGRITY Trial (ACTRN12623000127606) and delivers multi‐domain intervention in an intergenerational environment. The pilot will recruit 40 community‐dwelling adults aged 65+, with no cognitive impairment, and 40 pre‐school children aged 3‐6. Four preschools will be cluster‐randomised into two arms. The intervention arm will receive the GenWell‐BRAVE program, involving physical, social, and cognitive activities whilst building PMH skills through PPI. Participants will receive the GenWell‐Brave program at the preschool for 20 weeks, for 2 hours each week. The active control arm will receive a comparable program without PPI. Pre‐post questionnaires (at baseline and end of program), qualitative interviews, and systematic behavioural observations will be used to evaluate the program.

**Result:**

The primary outcome is comparison, between the trial arms, of change in cognitive score (NIH Toolbox Cognition Battery). Additional outcomes include change in PMH (MHC‐SF), dementia risk score (LIBRA‐2), and emotional regulation (DERS‐SF). Feasibility will be evaluated through measures of adherence, retention, implementation fidelity, and acceptability (TFA‐Q).

**Conclusion:**

We anticipate GenWell‐BRAVE will show adequate feasibility. We expect to establish the value of PPI in multi‐domain intervention, through providing preliminary evidence that a program which co‐intervenes on PPI has superior effectiveness in maintaining cognition.

